# Transitional fetal hemodynamics and gas exchange in premature postpartum adaptation: immediate vs. delayed cord clamping

**DOI:** 10.1186/s40748-019-0100-1

**Published:** 2019-04-12

**Authors:** Berk Yigit, Ece Tutsak, Canberk Yıldırım, David Hutchon, Kerem Pekkan

**Affiliations:** 1 Department of Biomedical Engineering, Carnegie Mellon University, Pittsburgh, PA USA; 2 Department of Biomedical Engineering, Politecnico di Milano, Milan, Italy; 3 Department of Mechanical Engineering, Boğaziçi University, Istanbul, Turkey; 4 Emeritus Consultant Obstetrician, Memorial Hospital, Darlington, UK; 5 Department of Mechanical Engineering, Koç Univeresity, Istanbul, Turkey

**Keywords:** Fetal hemodynamics, Premature birth, Cord clamping, Postnatal adaptation, Cardiovascular system, Lumped parameter modelling, Neonatal resuscitation

## Abstract

**Background:**

Recent studies suggest that delayed cord clamping (DCC) is advantageous for achieving hemodynamic stability and improving oxygenation compared to the immediate cord clamping (ICC) during fetal-to-neonatal transition yet there is no quantitative information on hemodynamics and respiration, particularly for pre-term babies and fetal disease states. Therefore, the objective of this study is to investigate the effects of ICC and DCC on hemodynamics and respiration of the newborn preterm infants in the presence of common vascular pathologies.

**Methods:**

A computational lumped parameter model (LPM) of the placental and respiratory system of a fetus is developed to predict blood pressure, flow rates and oxygen saturation. Cardiovascular system at different gestational ages (GA) are modeled using scaling relations governing fetal growth with the LPM. Intrauterine growth restriction (GR), patent ductus arteriosus (PDA) and respiratory distress syndrome (RDS) were modeled for a newborn at 30 weeks GA. We also formulated a “severity index (*SI*)” which is a weighted measure of ICC vs. DCC based on the functional parameters derived from our model and existing neonatal disease scoring systems.

**Results:**

Our results show that transitional hemodynamics is smoother in DCC compared to ICC for all GAs. Blood volume of the neonate increases by 10% for moderately preterm and term infants (32–40 wks) and by 15% for very and extremely preterm infants (22–30 wks) with DCC compared to ICC. DCC also improves the cardiac output and the arterial blood pressure by 17% in term (36–40 wks), by 18% in moderately preterm (32–36 wks), by 21% in very preterm (28–32 wks) and by 24% in extremely preterm (20–28 wks) births compared to the ICC. A decline in oxygen saturation is observed in ICC received infants by 20% compared to the DCC received ones. At 30 weeks GA, SI were calculated for healthy newborns (1.18), and newborns with GR (1.38), PDA (1.22) and RDS (1.2) templates.

**Conclusion:**

Our results suggest that DCC provides superior hemodynamics and respiration at birth compared to ICC. This information will help preventing the complications associated with poor oxygenation arising in premature births and pre-screening the more critical babies in terms of their cardiovascular severity.

## Background

The circulatory system of a newborn undergoes drastic transitions to adapt to the *ex utero* life, during which respiratory function is transferred from placenta to lungs. A smooth and event free transition is important for infant’s well-being and constitutes grounds for a healthy pediatric development. Although being very common, it is recommended to avoid immediate cord clamping (ICC) as its harmful effects for neonates are being documented in recent bodies of work [1, 2]. Our recent work elucidated the role of hemodynamics in circulatory transition from fetal-to-neonatal life in “term” neonates [3]. An abrupt removal of the placental circulation via ICC resulted in a lower cardiac output, a lower organ blood flow mediated by a decreased cardiac preload related to hypovolemia, and neonatal hypoxia when the cord is clamped before ventilation is established [4]. These quantitative findings are supported by the clinical studies performed during or immediately after birth in which delayed cord clamping (DCC) was found to be improving the early oxygenation [5], cardiac output [6], blood volume [7], in human neonates, and hemodynamic stability in fetal lamb studies [8] compared with ICC. Consequently, it has been observed that DCC has a lower incidence of bradycardia [8], iron deficiency [9] and provides an increased hematocrit [10] of the neonate during the early developmental phase. A reported adverse neonatal effect of DCC in term babies by The American College of Obstetricians and Gynecologists Committee is increase of jaundice requiring phototherapy, without any adverse maternal effect [11]. On the other hand, while systematic reviews of randomized controlled trials in babies born claimed DCC reduced the incidence of intraventricular hemorrhage [6, 10], more recent ones including meta-analysis concluded that effect of DCC on reducing all grades of intraventricular hemorrhage is no longer significant [12, 13].

In this study, we investigate the impact of cord clamping in premature birth at 20 weeks’ gestation through 40 weeks of gestation in normal and compromised infants. We use our computational lumped parameter model (LPM) of the fetal circulatory system that has been developed and validated previously for the investigation of transitional hemodynamics and gas exchange at birth of a healthy term infant [3]. By expanding the model with cardiovascular scaling functions [14], we are able to simulate the transitional hemodynamics and gas exchange during birth for babies that are born at gestational ages ranging from 20 to 40 weeks. The model is further tuned for simulating common perinatal diseases that affect a large number of premature births such as respiratory distress syndrome (RDS), patent ductus arteriosus (PDA), and fetal growth restriction (GR). RDS can be caused by the insufficient production of surfactants by neonatal lungs, which causes the alveoli to collapse during breathing. That insufficiency is compensated by the more disseminated use of surfactants and the continuous positive airway pressure (CPAP) treatment in recent clinical applications to intervene the RDS [15, 16]. PDA, which is the persistent patency of ductus arteriosus, is a common type of cardiovascular problem with a high incidence rate of 8 in 1000 of premature births [17]. With the decrease in PVR, PDA leads to large left-to-right shunting through the DA. Since pulmonary over-circulation is observed due to this shunt, excessive blood volumes are delivered to the lungs. Therefore, the left ventricle/left atrium recieves the increased pulmonary venous, which may lead to congestive heart failure. In GR, the placenta is underdeveloped due to the large placental vascular resistance (PlVR) and the fetal cerebral circulation adapts to this condition by lowering the cerebral vascular resistance (CVR) to increase the distribution of blood to the brain, which is called the ‘brain-sparing effect’ [18, 19]. Quantifying the transitional dynamics and the effects of cord clamping is paramount for advancing our understanding of the perinatal diseases and for improving the clinical management of problematic premature birth.

## Methods

### Hemodynamic model

**Fig. 1:**
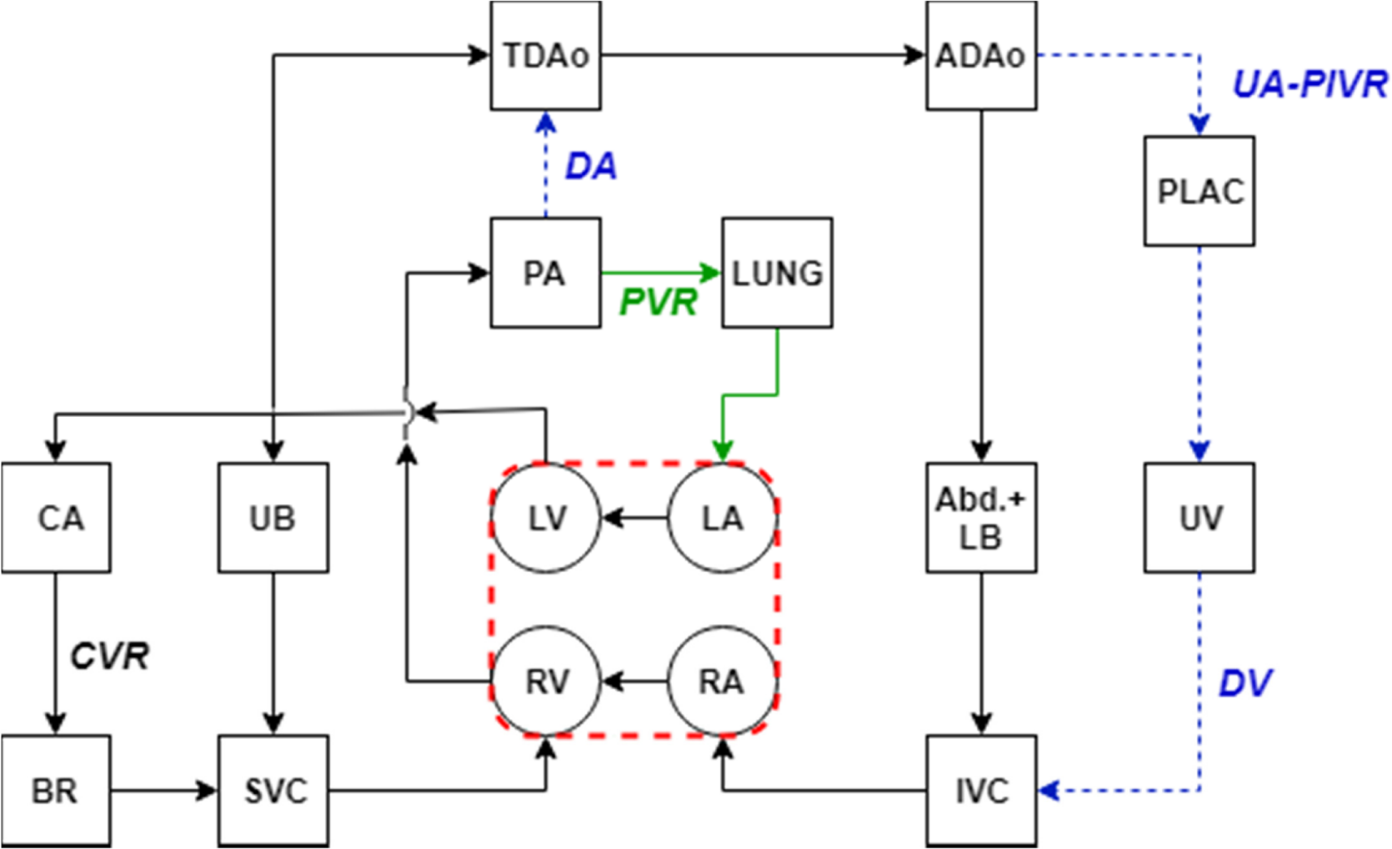
LPM network schematics of the transitionary fetal cardiovascular circuit. Connecting lines represent the arterial, capillary and venous resistances and compartments represent the compliant chambers of the corresponding elements. Arrows describe the direction of flow in vessels and/or valves. *Green* colored line represents the connections that open during the fetal-to-neonatal transition and the *blue* one stands for the connections that close. LV: left ventricle, LA: left atrium, RV: right ventricle, RA: right atrium, PA: pulmonary artery, CA: carotid artery, UB: upper body, BR: brain, SVC: superior vena cava, TDAo: thoracic descending aorta, LUNG: lungs, ADAo: abdominal descending aorta, PLAC: placenta, UA-PlVR: umbilical artery-placental vascular resistance, Abd. + LB: abdomen+lower body, UV: umbilical vein, IVC: inferior vena cava, DA: ductus arteriosus, PVR: pulmonary vascular resistance, CVR: cerebral vascular resistance, DV: ductus venosus

LPM is a practical way of investigating the pulsatile hemodynamics by modeling the whole circulation as an analogous electric circuit that consists of resistances and compliances. Circulation network can be traced starting from the left ventricle (LV) and blood flows through the vessels of systemic and pulmonary circulation. In our LPM network, while the compliance chambers were used to represent vascular beds, the resistances connecting these compartments model hydrodynamic energy loss due to viscous blood flow (Fig. 1). Using our neonatal and pediatric circulatory LPM framework [3], we constructed a representative fetal circuit inspired from the earlier network designs by Pennati et al. [20] and Sa-Couto et al. [21]. This model is described in detail and validated for the transitional hemodynamics from fetal to neonatal life of a healthy term infant (~ 40 weeks in gestation) in our recent paper (Fig. 1) [3]. Both preterm and term fetuses are modeled as normal for gestational age.

To represent the earlier preterm stages of pregnancy, the term model is scaled to earlier stages in fetal development with allometric and similitude scaling principles [14]. Eq. 1 is a power law function that represents the changes in vascular parameters (Y), such as resistances (R), compliances (C) and volumes (V) with respect to fetal growth captured by the fetal weight (W) [22]:

1 $$ {Y}_{GA}/{Y}_{40}={\left({W}_{GA}/{W}_{40}\right)}^b $$

where W_GA_ is the fetal weight and, Y_GA_ is the value of a physiological parameter (e.g. R, C, V) at a given gestational age GA. W_GA_ is determined from the empirical formula log_10_*W*_*GA*_ = 0.2508 + 0.1458 × *GA* − 0.0016 × *GA*^2^ [23]. Y_40_ represents reference values for the circulatory parameter at 40 weeks gestation, which are based on the term fetal/neonatal LPM analysis. The exponential coefficient *b* in the power law equation (Eq. 1) is determined from scaling relations for each circulatory parameter (Table 1) [14, 22].

**Table 1:** Organ specific exponential coefficient *b,* for vascular resistance (R) and compliance (C) parameters

	Common Vessels	Common Organs	Brain	Lungs	Placenta	Foramen Ovale	Ductus Arteriosus	Ductus Venosus
R	−1.00	−0.66	−0.77	− 0.86	−0.45	− 0.60	− 1.00	−0.55
C	1.00	1.00	1.20	1.30	0.56			

The exponential coefficients reported in Table 1 are assumed for the resistances and compliances associated with the corresponding organ vascular beds, and common arteries and veins. Special cases are included for organs (brain, lungs, placenta) and shunts (Foramen Ovale, Ductus Arteriosus, Ductus Venosus) that show distinctive growth patterns.

Finally, the fetal heart rate (HR) is varied according to the empirical linear function, *HR* =  − 0.5 ∗ *GA* + 160 (min^− 1^) covering the gestation period between 20 to 40 weeks [24].

### Gas exchange model

Gas exchange model is described in detail and validated for a healthy term fetus/neonate in our recent paper [3]. In all gestational ages, we assumed a fetal O_2_ consumption of 8 ml-O_2_.min^− 1^.kg^− 1^ (for the organ distribution of metabolic consumption, please refer to the [17]). For scaling the model towards earlier gestational ages, we used clinical reference ranges for the necessary physiological variables, such as the fetal hemoglobin concentration [3].

### Circulatory and respiratory transition at birth

Shunt transitions after birth are modeled by the time-dependent constriction of ductus arteriosus (DA), ductus venosus (DV), umbilical arteries (UA) and umbilical vein (UV) that is simulated with an increase in the hemodynamic resistance of the respective vessels. UA and UV are constricted immediately and simultaneously in ICC. On the other hand, in DCC, UA is transiently constricted and UV is left open reflecting the postnatal transition without clinical intervention. Transitions in the pulmonary circulation are modeled as a decrease in PVR by 8-fold, reflecting the expansion of pulmonary vasculature. The rates of vascular transition were determined from fetal lamb experiments [25] and by matching the systemic and pulmonary arterial blood pressures from simulations to ones that were obtained in human after birth with catheterization [26, 27]. We assumed that the transition rates and their durations are similar in preterm and term neonates. Equations of transition modeling are explained in our previous publication [3].

We investigated the hemodynamics and gas exchange for the disease states of RDS, PDA and GR through idealized models, as in normal term infants. To reflect the effects of RDS in the model, pulmonary vascular resistance and ductus arteriosus resistance are unchanged from their fetal values for the whole duration of postnatal adaptation period. Similarly, PDA is modeled by keeping the DA resistance unchanged during the transition but PVR decreases by 8-fold just as normal transition. We increased the placental vascular resistance by 50% and reduced the cerebral vascular resistance by 50% for the GR model to represent the underdeveloped placental circulation and the accompanying brain sparing effect [28], but the transition follows the course of an otherwise healthy circulation. Other patient-specific scenarios employing different model parameters can be studied similarly.

### Severity index

Based on our previous investigation focusing on healthy term babies [3], we have selected the cerebral blood flow rate, systemic arterial blood pressure, pulmonary blood pressure, neonatal blood volume and cerebral oxygen saturation (ScO_2_) as the severity metrics. Large differences in these parameters between ICC and DCC would favor DCC over ICC. To obtain a compact measure which represents the combined impact of these various metrics, we have formulated a severity index (*SI*), which integrates these metrics of cardiovascular performance into a single severity score. For each metric *δ*, the relative difference in *δ* between ICC and DCC is calculated as a percent difference for preterm births, *PD*_*preterm*_ (GA < 40wk, Eq. 2), which is then normalized with the percent difference for term births, *PD*_*term*_ (GA = 40 wk., Eq. 3). *SI* is obtained as the weighted sum of normalized *PD* s where each *PD* has a weighting factor *w* associated with it, as shown in Eq. 4. In the present study, the weighting factors for all indices are equally distributed, while satisfying ∑*w* = 1.

2 $$ {PD}_{preterm}={\left\Vert \frac{\delta_{ICC}-{\delta}_{DCC}}{\delta_{DCC}}\right\Vert}_{preterm} $$

3 $$ {PD}_{term}={\left\Vert \frac{\delta_{ICC}-{\delta}_{DCC}}{\delta_{DCC}}\right\Vert}_{term} $$

4 $$ SI=\sum w.\left(\frac{PD_{preterm}}{PD_{term}}\right) $$

SI can be interpreted as the impact of cord clamping in premature birth at a given age relative to its impact in a term birth. All *δ* use the absolute values of the corresponding hemodynamic variable obtained from the model at the end of postnatal transition with ICC and DCC, except for the *δ* for ScO_2_, which takes the *δ*_*ICC*_ value as the lowest ScO_2_ observed during the transition in ICC and the *δ*_*DCC*_ value from DCC at the same time as *δ*_*ICC*_ was recorded. The intention behind this choice is to capture the severity of the hypoxia observed during the early transitional period in ICC.

## Results

### Validation of the premature hemodynamic model through the gestation

We assessed the validity of the preterm model by comparing model outputs with the clinical reference ranges for flow rates, Doppler velocity waveform indices, arterial blood pressures and umbilical blood gas measurements. During gestation, fetal combined cardiac output (CCO) increases proportional to the fetal weight and the reported CCO per fetal weight is in the range of 400–425 ml.min^− 1^.kg^− 1^ [18, 29–31]. Simulated CCO is on the average 450 ml.min^− 1^.kg^− 1^, which was in the range of clinical reference values as displayed in Fig. 1a. Simulated changes in organ blood flow and shunt flow distributions during gestation are displayed in Fig. 1b. In the simulations, the ratio of right- to left-ventricular output increases from 1.2 at 20 weeks gestation to 1.33 at 40 weeks gestation, which is also in agreement with the values reported in the literature [29, 30, 32]. The fraction of fetal CCO directed to the placenta decreases from 30% at 20 weeks gestation to 20% at 40 weeks (same range as reported in [18]), whereas the fraction directed to the pulmonary circulation increases from 20 to 30% (same range as reported in [29]). We found good resemblance of the simulated cardiac output distribution to organs with that were measured in fetal lamb experiments, such as an increase in the CCO distribution to the brain with advancing gestation [33]. Simulated arterial pulsatile blood pressures are in the range of blood pressure estimations from human fetal Doppler velocity waveforms in [34] as displayed in Fig. 1c. Pulsatile hemodynamics is assessed by qualitative comparisons of simulated flow waveforms with their clinically sampled human fetal Doppler velocity waveforms sampled at various major sites and their derived indices. Pulsatility index (PI) for different vessels is calculated from the simulations using $PI=\frac{Q_{max}-{Q}_{min}}{Q_{mean}}$, where *Q* is the flow rate in the respective vessels, and *Q*_mean_ is the cardiac-cycle averaged flow rate. Simulated and clinically reported PIs for different fetal vessels [24, 35–38] are compared in Fig. 1d. Atrioventricular E/A ratios that are obtained from simulations and that are reported in clinical measurements [35, 39] are similarly compared in Fig. 1e.

Papers based on clinical trials and fetal lamb experiments report that the fetal blood PO_2_ decreases as the gestation advances [40, 41], but the umbilical venous O_2_ concentration of the fetal blood remains constant [42]. According to Link et al., PO_2_ at delivery (range, 29–42 weeks) measured from the umbilical arteries is lower in term infants (40 ± 2 weeks) at 19.2 (±8.6) mmHg compared to preterm infants (33 ± 3 weeks) at 23.9 (±5.3) mmHg. Similarly PO_2_ measured from the umbilical vein is 24.6 (±6.5) mmHg in term infants compared to 29.4 (±8.6) mmHg in preterm infants [41]. Simulated variations in PO_2_ during the course of gestation are displayed in Fig. 1f.

### Hemodynamics and gas exchange in normal premature birth

**Fig. 2:**
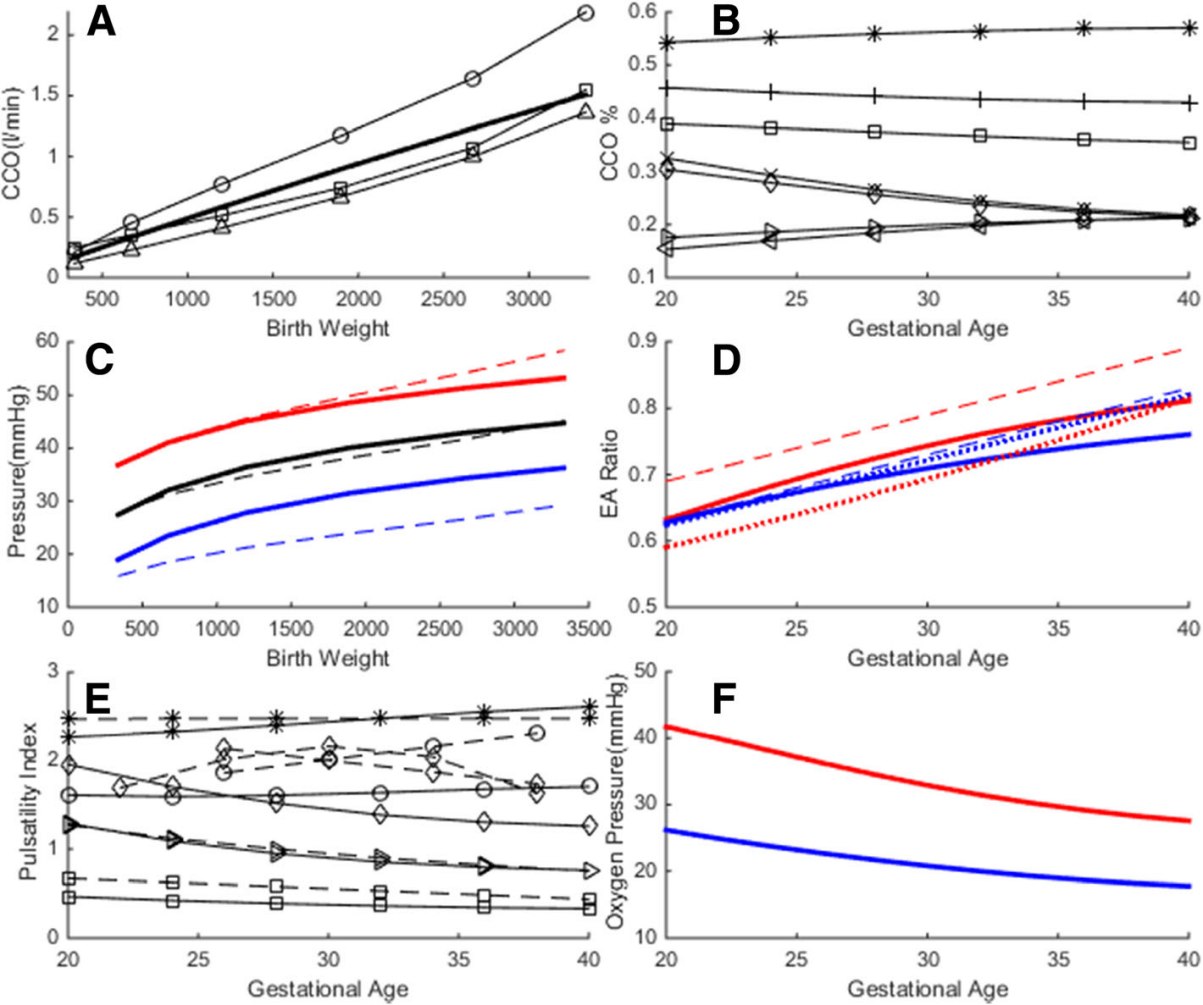
Validation of the computational fetal hemodynamics/gas exchange model for the range of gestational ages under consideration. Please note that the model is rigorously validated for term babies in Ref. [3]. **a** Combined cardiac output (CCO), solid line: simulated, circle: Rasanen et al. [29], square: De Smedt et al. [31], triangle: Kiserud et al. [18] **b**) Simulated organ flow distribution as percentage of CCO, asterisk: RVO, plus: LVO, square: DA, cross: PLAC, diamond: FO, right-triangle: CEB, left-triangle: PUL. **c** Aortic blood pressure. Solid: simulated, dash: clinical [27], red: systolic, black: mean, blue: diastolic. **d** E/A ratio. Red: mitral, blue: tricuspid, solid: simulated, dash: Hecher [35], dot: Kenny [39]. **e** Pulsatility index, solid: simulated, dash: clinical, asterisk: DA, diamond: MCA, circle: TAo, right-triangle: UA, square: DV. Clinical values are taken for DA from Mielke et al. [36], MCA from Ebbing et al. [38] and Ferrazzi et al. [24], TAo from Ferrazzi et al. [24], UA from Acharya et al. [37], DV from Hecher et al. [35]. **f** Simulated oxygen partial pressure in umbilical vessels, red: UV, blue: UA. RVO: right ventricular output, LVO: left ventricular output, DA: ductus arteriosus, PLAC: placenta, FO: foramen ovale, CEB: cerebral, PUL: pulmonary, MCA: middle cerebral artery, TAo: thoracic aorta, DV: ductus venosus, UA: umbilical artery, UV: umbilical vein

We performed simulations of circulatory and respiratory changes during fetal to neonatal transition with ICC and DCC of premature infants with gestational ages ranging from 20 to 40 weeks. Due to the prevention of placental transfusion, infants that underwent ICC procedure suffered a loss of 10% (32 wk. < GA < 40 wk) and 15% (20 wk. < GA < 32 wk) in blood volume compared to preterm infants, which underwent DCC. The decreased blood volume in ICC resulted lower cardiac output, a lower organ blood flow and lower blood pressure: cardiac output and the arterial blood pressure were reduced by 17% in term (36–40 wks), by 18% in moderately preterm (32–36 wks), by 21% in very preterm (28–32 wks) and by 24% in extremely preterm (20–28 wks) cases. This reduction in cardiac output in ICC was related to a loss in the postnatal cardiac preload and hypovolemia, as seen for the case of pulmonary blood flow in Fig. 2b [3]. Reduction in organ blood flows were proportional to the reduction in cardiac output at all gestational ages.

Due to the sudden removal of placental respiratory path in ICC, all infants suffered a temporary hypoxia during the early postnatal transition period (Fig. 2a). In the simulations, the lowest recorded ScO_2_ was 26.3% on average for full term infants, and the hypoxia worsened as the lowest ScO_2_ dropped by 25% of its fetal value in premature cases (GA = 30 wk). Respiratory transition was smooth with DCC in all cases and the SO_2_ levels always remained above the fetal range after birth. SO_2_ levels at the end of transitions did not differ significantly between DCC and ICC cases.

**Fig. 3:**
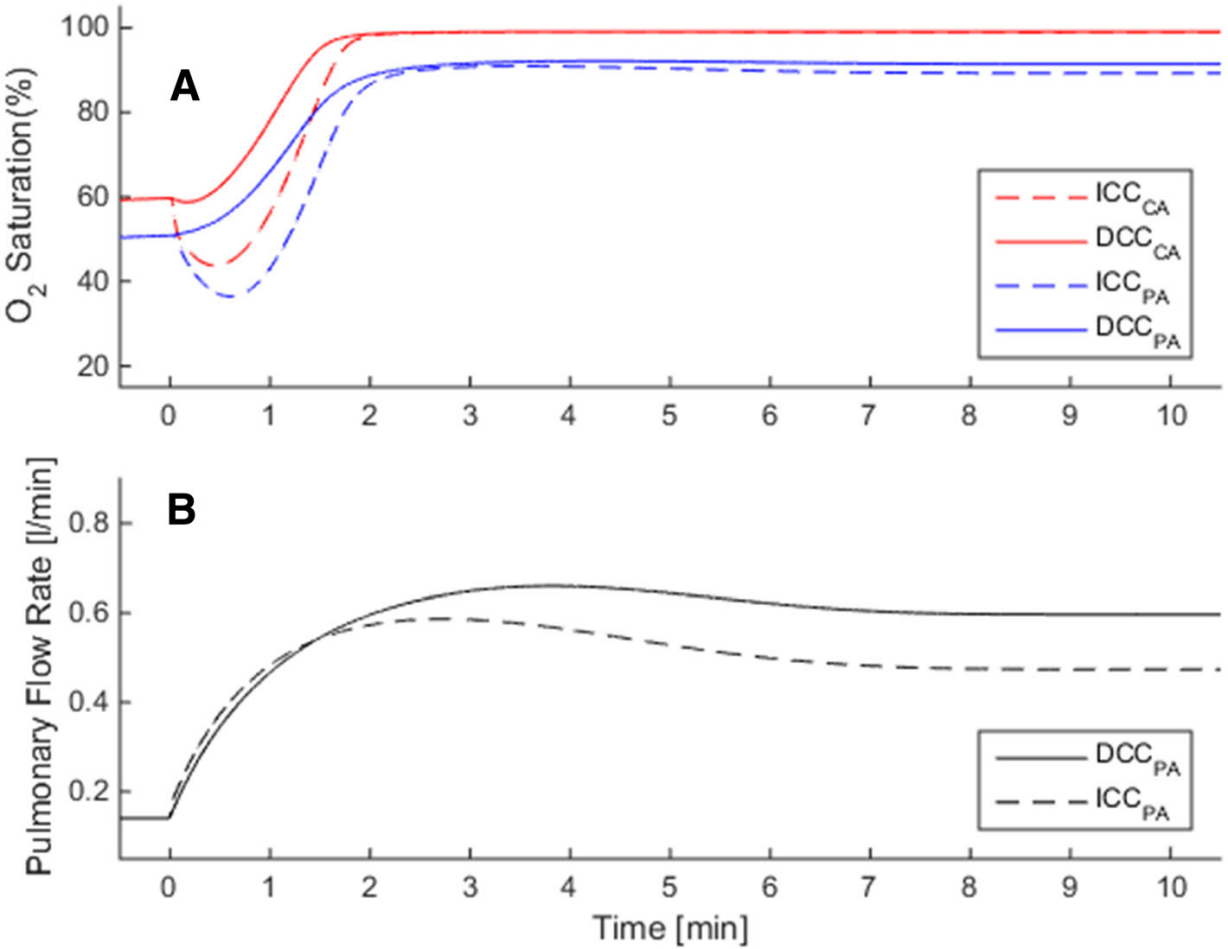
Circulatory and respiratory changes during post-natal transition in a premature but otherwise healthy case (GA = 30 wk). Plots compare transitional dynamics in delayed cord clamping (DCC, solid lines) against immediate cord clamping (ICC, dashed lines). Early oxygenation is adversely affected in ICC due to the sudden loss of placental respiration, as seen in **a** for CA and PA. Organ flow rates are lower in ICC compare to DCC by 21%, as seen in (**b**) for the pulmonary circulation. CA: carotid arteries, PA: pulmonary arteries

Severity indices for premature cases where gestational ages ranged from 20 weeks to 40 weeks for term, are calculated using the methodology that is explained in severity index part. SI is 1.0 for a term infant as expected, and increases with smaller gestational ages reaching 1.18 at 30 weeks’ gestation and reaching 1.5 at 20 weeks gestation (Fig. 3).

### Hemodynamics and gas exchange in premature birth with impaired circulation

**Fig. 4:**
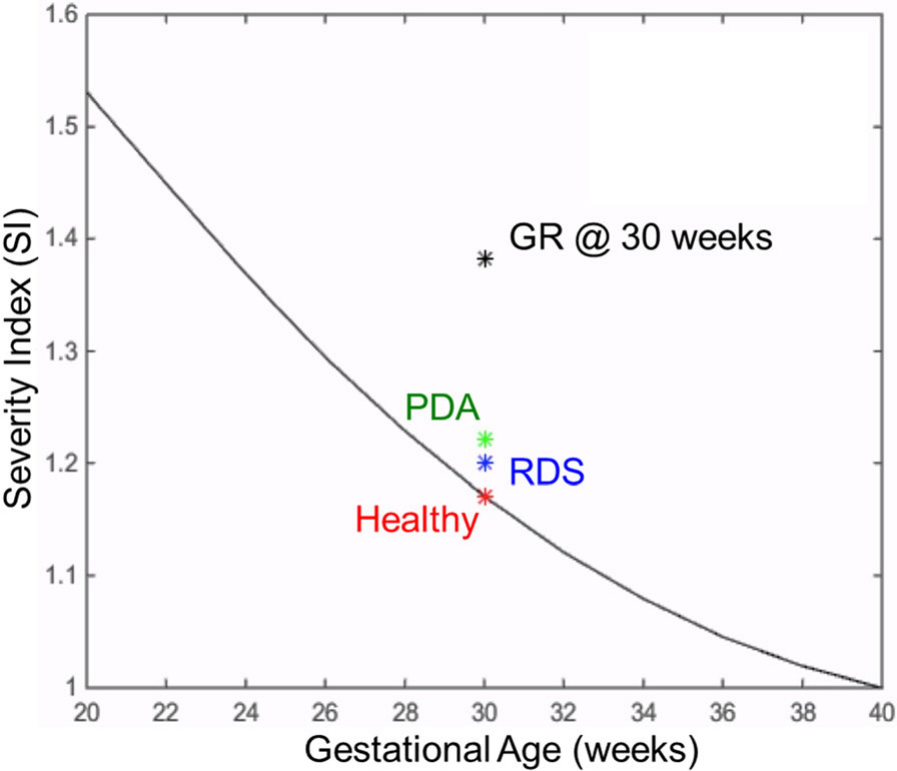
The solid line shows the Severity Index (SI) changes throughout the gestational period, for premature but otherwise healthy infants. SI is calculated from Eq. 4, in which the immediate cord clamping (ICC) hemodynamics is compared to the delayed cord clamping (DCC). ICC results an increasingly adverse impact on circulatory and respiratory adaptation during the early postnatal transition period for preterm infants. The plot also displays SI values of the three diseased preterm cases investigated in this study, at the 30th week gestational age (labeled with asterisk symbol). These disease states are respiratory distress syndrome (RDS) in blue, patent ductus arteriosus (PDA) in green, and growth restriction (GR) in black are plotted alongside the normal premature SI curve for comparison

**Fig. 5:**
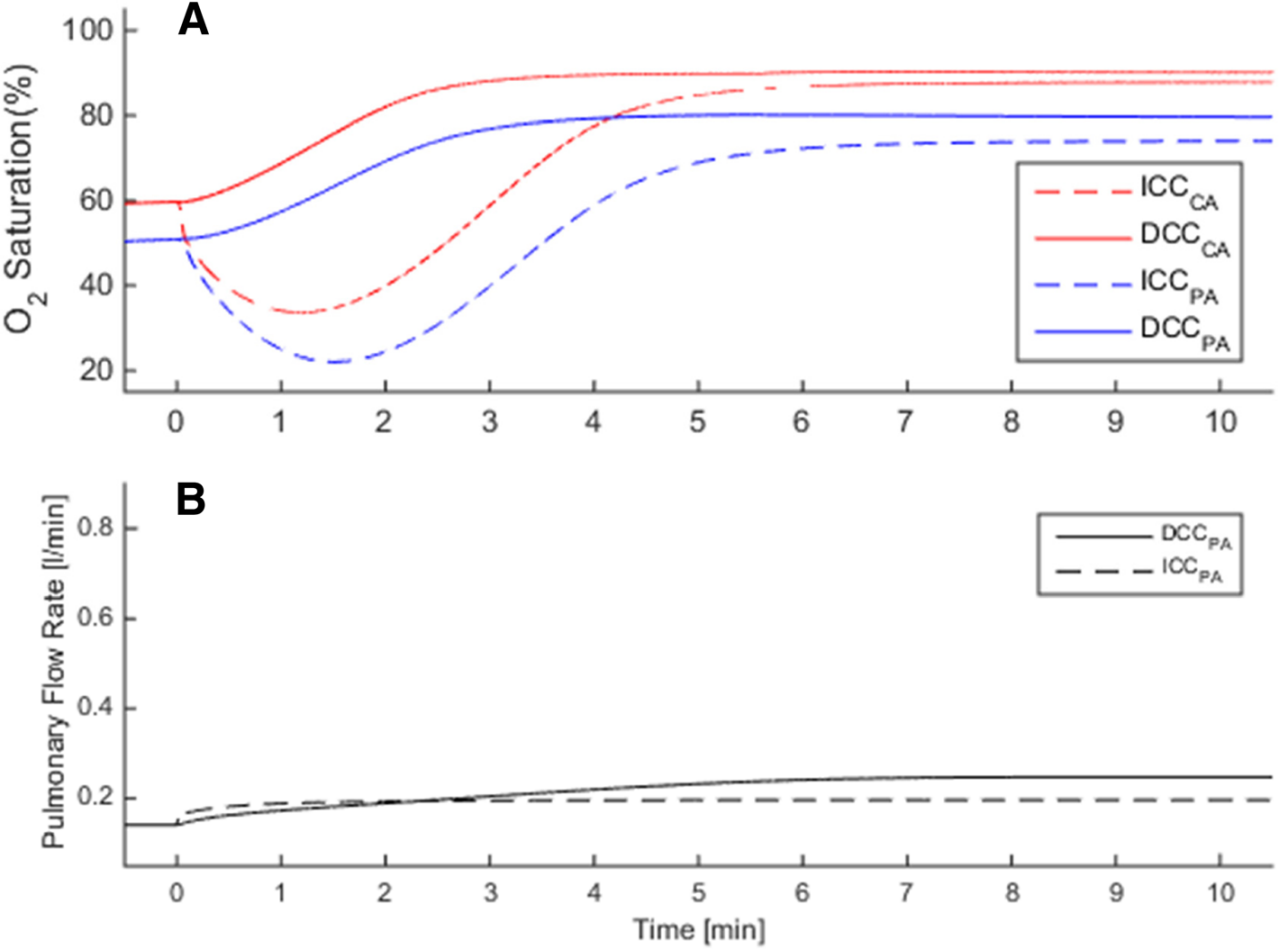
Circulatory and respiratory changes during post-natal transition in a premature case (GA = 30 wk) with respiratory distress syndrome (RDS), in which pulmonary vascular resistance does not fall and ductus arteriosus remains patent. Plots compare transitional dynamics in delayed cord clamping (DCC, solid lines) against immediate cord clamping (ICC, dashed lines). Adverse effect of ICC on early oxygenation is more profound and prolonged due to RDS compared to normal premature birth, as seen in **a** for CA and PA. Pulmonary flow rate in ICC is lower compared with DCC by 21% as seen in **b**. Axis ranges are the same as Fig. 3 in order to facilitate comparisons between normal and RDS cases. CA: carotid arteries, PA: pulmonary arteries

In RDS, neonatal circulation cannot transition fully to the biventricular configuration since PVR remains high and a patent DA is observed. Following, a large right to left shunt is observed and pulmonary blood flow remains very low. Contributed by the increase in peripheral vascular resistance due to the removal of the low resistance placental vasculature, the cardiac output remains significantly lower than the normal premature birth condition (CCO = 0.68 L/min for ICC and 0.86 L/min for DCC). In DCC, we observe that pulmonary blood flow is slightly higher compared to ICC (Fig. 4a), but the major improvement is observed in the oxygenation. In the simulations, postnatal transition with ICC in RDS is marked with a profound and prolonged decrease in arterial and cerebral SO_2_ during the early adaptation period as seen in Fig. 5a. ScO_2_ attains a minimum of 20% and does not go above its fetal value before the first 5th minute in birth. In DCC, ScO_2_ remains above the fetal value during the whole postnatal period as the placental respiration is maintained along with contribution from the pulmonary respiration. In the late transitional period during which the placental circulation is completely removed, we observe that the ScO_2_ and pulmonary arterial SO_2_ are higher in DCC compared to ICC since the increased blood volume led to an increased oxygen capacity of the circulation.

**Fig. 6:**
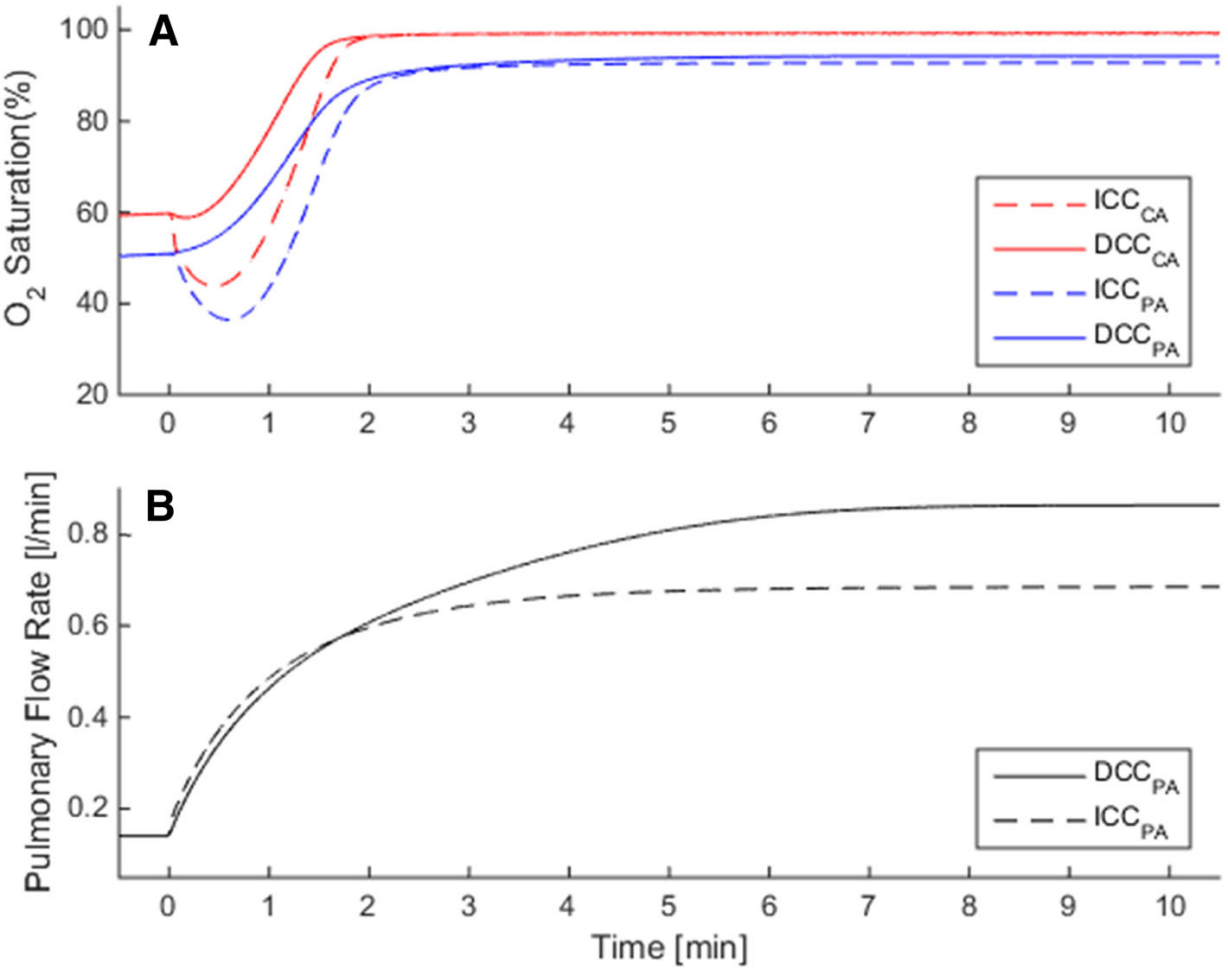
Circulatory and respiratory changes during post-natal transition in a premature case (GA = 30 wk) with patent ductus arteriosus (PDA), in which pulmonary vasculature does relax but ductus arteriosus remains patent. Plots compare transitional dynamics in delayed cord clamping (DCC, solid lines) against immediate cord clamping (ICC, dashed lines). Comparison of oxygenation in ICC with DCC is seen in **a** for CA and PA. Pulmonary flow rate is higher than normal premature birth by 44% as seen in **b**. Axis ranges are the same as Fig. 3 in order to facilitate comparisons between healthy and PDA cases. CA: carotid arteries, PA: pulmonary arteries

In the PDA simulations, PVR decreases but DA does not constrict as expected in a healthy transition. Consequently, a large left-to-right shunt is observed and pulmonary blood flow is much larger than the normal range. This shunt is mainly maintained by an increased left ventricular output (LVO), which constitutes 60% of the CCO (LVO = 0.68 L/min in ICC and = 0.87 L/min in DCC). This condition results with a larger stroke volume and more work input by the left ventricle. Likewise, the right ventricle needs to operate against a larger afterload due to an increased pulmonary arterial blood pressure (34/42 mmHg in ICC/DCC with PDA compared to 23/30 mmHg (ICC/DCC) in normal premature birth at 30th week gestation). The increase in LVO does not improve the organ blood flow rates, which are ~ 10% lower than the normal premature case when complications other than prematurity are absent. The effect of ICC is similar to the normal premature case, in which the sudden removal of placental respiration causes the arterial and cerebral SO_2_ to decrease in the early transition period (Fig. 6a).

**Fig. 7:**
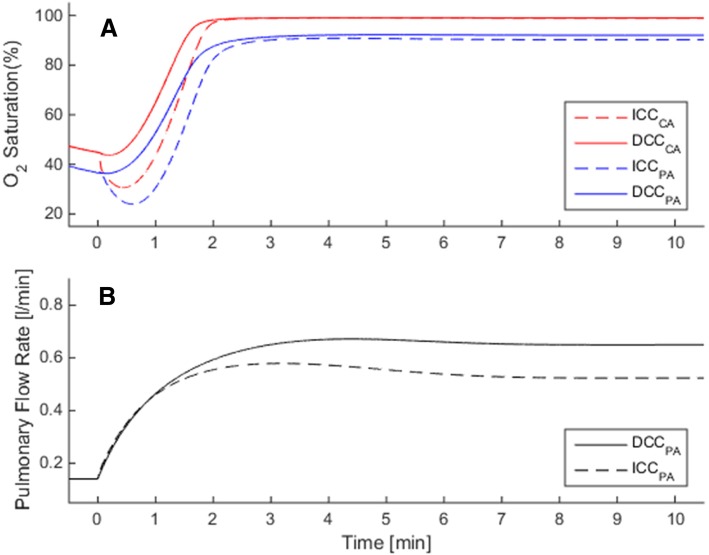
Circulatory and respiratory changes during post-natal transition in a premature case (GA = 30 wk) with fetal growth restriction (GR), due to which placental circulation is underdeveloped (placental vascular resistance is 50% higher compared to normal premature) and cerebral circulation is under brain-sparing effect (cerebral vascular resistance is 50% lower compared to normal premature). Plots compare transitional dynamics in delayed cord clamping (DCC, solid lines) against immediate cord clamping (ICC, dashed lines). Adverse effect of ICC on early oxygenation is more profound due to GR, where the cerebral oxygen saturation (ScO_2_) falls to ~ 35% as seen in **a**. Comparison of pulmonary flow rates in ICC with DCC is shown in **b**. Axis ranges are the same as Fig. 3 in order to facilitate comparisons between healthy and GR cases. CA: carotid arteries, PA: pulmonary arteries

Finally, in GR, the placental circulation is underdeveloped and it is balanced by the brain-sparing effect. The fetal SO_2_ levels are lower than the healthy circulation and ScO_2_ falls to critically low levels in case of ICC (lowest ScO_2_ ~ 30%, Fig. 7a). Pulmonary blood flow in GR is affected similarly to the healthy case simulations by respective cord clamping scenarios (Fig. 7b).

## Discussion

Established benefits of DCC for term infants, directed the recent investigations towards establishing the feasibility and safety of DCC in premature birth cases. A number of pioneering clinical trials that were conducted recently suggested that DCC does provide hematological, circulatory and respiratory advantages compared to ICC for very preterm infants (< 32 weeks’ gestation) [43, 44], and ICC should be avoided unless absolutely necessary [2], for example, such as in nuchal cord or in case of failure of placental function [45]. These studies provided solid insights on the applicability of DCC in premature births. Yet, until present, a detailed investigation of the variability of the hemodynamic and respiratory severity of ICC depending on the gestational age at the time of birth has been unavailable in the clinical literature. Our model results suggest that DCC does not represent any noticeable disadvantage over ICC regarding hemodynamic and respiratory functions. On the contrary, our model demonstrated certain adverse effects of ICC including decreased cardiac output, blood pressure, hypovolemia, and temporary hypooxygenation during early perinatal period. Based on our results, prematurity intensified these adverse effects of ICC since a higher fraction of fetal blood is contained within placenta with smaller gestational ages. Premature newborns have underdeveloped lungs and smaller hemoglobin reserves, which results in difficulty of breathing and prevalence of respiration related complications at birth. In these cases, our results suggest that sustaining placental perfusion in the immediate postnatal duration by DCC is advisable for maintaining the blood oxygen saturation as high as possible.

We performed a precursory investigation of the impact of umbilical cord clamping practices on hemodynamics and respiration when perinatal cardiovascular disease is present. *SI* for the three disease cases are plotted in Fig. 3, which shows that the most severe adverse effects of ICC are felt in RDS, then in GR, and lastly in PDA. Critically low SO_2_ levels combined with the low ventricular outputs makes RDS the most critical disease among the considered diseases. Severity of the newborn’s condition will more likely depend on the severity of the disease itself (mild/severe) and multiple diseases might be presented at the same time. Mapping severities of a broader spectrum of disease scenarios are left for future work, for which we laid the groundworks in this study.

We developed and extensively validated a LPM of the circulatory and respiratory system of the premature infant during the transition from fetal to neonatal life over a wide range of gestational ages. For clinical decision-making and preparation prior to delivery, computer-aided tools can be used for on-site prediction of the hemodynamic effects of cord clamping on a patient-specific basis. For this purpose, LPM provides a versatile framework, which can be adapted to patient-specific cases using echocardiographic measurements or using morphological scaling relations with sonographic organ size measurements [28].

Delivery planning and the prediction of patient-specific severity of perinatal diseases at the neonatal intensive care unit is a challenging task that require significant amount of obstetrician’s time and experience. Premature births present an additional challenge since blood pressure, cardiac output, blood volume, as well as cardiac output distribution vary with gestational age and infant size at birth. We formulated *SI* for grouping multiple cardiorespiratory performance factors in a single severity score with the aim of facilitating the interpretation of the cardiovascular performance of the neonate during postnatal cardiovascular transition. Similar scoring methodologies have been developed and used for standardizing the assessment of the cardiovascular condition of the newborn infant [46] in intrauterine growth restriction [47], hydrops fetalis [48] and congenital heart defects [49]. A distinctive feature of the *SI* is that our formulation targets to eliminate the bias of infant size and age over the severity score, by normalization of patient-specific measures with a reference value. For clinical adoption, *SI* will be further refined by choosing the weighting factors and selecting the most critical hemodynamic parameters through the input of clinicians, which is left for future work.

While the present approach faithfully incorporates the fundamental fetal hemodynamics and disease states of the fetus, it still has a few limitations: firstly, since LPM is a reduced order mathematical model it does not include multi-dimensional flow effects, such as mixing at the intersections of vascular components (this contribution is indeed negligible in large vessels compared to vascular resistances). Furthermore, in the present LPM the inertia of the flowing blood is not incorporated as our previous experience indicates that these effects are minor for most compliant neonatal regimes. Likewise, we did not attempt to consider all disease variables and all clinical complications associated with GR, PDA and RDS. For example, cardiovascular changes with the use of respiratory support (surfactants and CPAP) were not considered during modeling the transition. In addition, different modifications of changes in lung compliance due to the antenatal use of corticosteroids or any other surfactant preparations were not also included to this model, which can be investigated in the future studies through this type of modeling approach.

## Conclusion

Our quantitative investigation concluded that ICC protocol results with circulatory and respiratory adverse effects in premature birth, at all gestational ages. These cardiovascular effects include hypovolemia accompanied with a reduction in cardiac output, cerebral and organ blood flow, and hypoxia due to clamping prior to the establishment of ventilation. The adverse effects of ICC intensify with increased prematurity and whenever RDS, PDA or GR is present. Our results suggest that ICC is particularly deteriorative when RDS is present, which stresses the importance of maintained placental perfusion when natural ventilation is not achieved successfully. ICC prevents the neonate in distress to receive the helping boost it needs when going through the drastic adaptations to ex utero life.

## Abbreviations

CCO: Combined cardiac output
CPAP: Continuous positive airway pressure
CVR: Cerebral vascular resistance
DA: Ductus arteriosus
DCC: Delayed cord clamping
DV: Ductus venosus
GR: Fetal growth restriction
HR: Heart rate
ICC: Immediate cord clamping
LPM: Lumped parameter model
LVO: Left ventricle output
PDA: Patent ductus arteriosus
PI: Pulsatility index
PlVR: Placental vascular resistance
PVR: Pulmonary vascular resistance
RDS: Respiratory distress syndrome
SI: Severity index
UA: Umbilical arteries
UV: Umbilical vein
